# Exploring the Dependence and Influencing Factors of Carbon Emissions from the Perspective of Population Development

**DOI:** 10.3390/ijerph182111024

**Published:** 2021-10-20

**Authors:** Kuokuo Zhao, Xuezhu Cui, Zhanhang Zhou, Peixuan Huang, Dongliang Li

**Affiliations:** 1 School of Management, Guangzhou University, Guangzhou 510006, China; zhaokuok@126.com (K.Z.); peixuanhuang123@163.com (P.H.); 2 School of Economics and Management, Tianjin Chengjian University, Tianjin 300384, China; zhouzhanh@163.com (Z.Z.); ldlessay@163.com (D.L.)

**Keywords:** decoupling, STTRPAT, carbon mitigation, population development index (PDI)

## Abstract

Working towards sustainable population development is an important part of carbon mitigation efforts, and decoupling carbon emissions from population development has great significance for carbon mitigation. Based on the construction of a comprehensive population development index (PDI), this study adopts a decoupling model to explore the dependence between carbon emissions and PDI across 30 Chinese provinces from 2001 to 2017. Then, the stochastic impacts by regression on population, affluence and technology (STIRPAT) model is used to investigate the impact of population factors on carbon emissions. The results show that the decoupling relationship between carbon emissions and PDI has experienced a transformation from expansive negative coupling to expansive coupling and then to weak decoupling at the national level, while some provinces have experienced the same evolutionary process, but the decoupling state in most provinces is not ideal. Sending talent to western provinces and developing low-carbon supporting industries will accelerate carbon decoupling. At the national level, incorporating environmental protection into the existing education system as part of classroom teaching could contribute to carbon decoupling.

## 1. Introduction

### 1.1. Importance and Motivation

With the continuous growth of population and carbon emissions in developing countries, the issue of sustainable population development has attracted attention. In the face of the complex climate change situation, implementing carbon mitigation and sustainable population development strategies to reduce the climate risks caused by carbon emissions has become the top priority of the Chinese government [1]. To realize the coordinated development of the economy, society, resources and the environment, China, the country with the largest population, has experienced the transformation from the family planning policy to the three-child policy and the population sustainable development policy. During the demographic transition period, changes in population dynamics and development patterns will undoubtedly affect China’s energy use and the resulting carbon emissions [2]. Meanwhile, as the world’s largest carbon emitter, China is considered a key player in the international effort to tackle climate change [3]. In order to reduce carbon emissions, the Chinese government has also proposed the goal of peaking carbon emissions by 2030 and achieving carbon neutrality by 2060 [4]. To achieve the goal of dual carbon emission reduction firstly means that carbon emissions can no longer increase with the social development; that is to say, carbon emission decoupling is the first step to achieve carbon neutrality [5]. Therefore, evaluating the degree of carbon emission decoupling is of great significance for promoting carbon emission reduction.

Decoupling research originally focused on how to balance the tension between economic growth and environmental pollution [6]. Although some studies have examined this link at the national, industry or sector level, the decoupling between carbon emissions and population development is still unclear because it is rarely discussed. Quantifying the decoupling states of carbon emissions from a multiperspective can guide more detailed emission reduction strategies [7]. To achieve population sustainability and low-carbon development, it is of great importance for the government to understand the decoupling state between carbon emissions and population factors and the influencing mechanism between them [8,9].

Moreover, there are significant disparities regarding energy demand, population size, residential income, urban–rural structure and population quality in Chinese provinces, which may lead to significant differences in the interaction between carbon emissions and population development [10]. An objective evaluation of regional population development will provide an important premise for exploring the decoupling the relationship between carbon emissions and population development. Therefore, China and its 30 provinces for which data are available are selected for this study, which may contribute to a better understanding of the dynamics of carbon decoupling.

### 1.2. Objective and Contribution

Investigating the dynamics of the decoupling of carbon emissions from population development and making recommendations that might facilitate the decoupling of carbon emissions are key objectives of this study. The specific objectives are shown below:

(1) Develop a comprehensive indicator to reflect the multifaceted aspects of population development;
(2) Explore the decoupling relationship between carbon emissions and population development;
(3) Identify the driving force of population development on carbon emissions to promote carbon decoupling.

This study can make the contribution to the existing knowledge from the followings: Firstly, as for the research perspective, this study focuses on the dependence of carbon emissions on population development, while previous studies focus more on decoupling from the perspective of economic development. Since the population effect has no less of an impact on carbon emissions than economic development [11,12], this study focuses on the decoupling of carbon emissions from population, which is a good extension of the existing studies on the decoupling of carbon emissions and may help to promote managers to focus on high-quality transition and the sustainable development of population, rather than only sustainable development at the economic level. Secondly, regarding the research scale, this study investigates the decoupling state between population development and carbon emissions at the national and provincial level. China is the world’s most populous country, with a population of more than 1.4 billion. Of the 30 provinces for which data are available, 20 have a population of more than 30 million in 2017 [13]. As an important contributor to carbon emissions, the study on the decoupling of China’s carbon emissions will not only help China’s energy conservation and emission reduction process, but also provides a reference for sustainable population development in other populous countries, such as India and other countries, and plays an important role in promoting climate change mitigation worldwide. Finally, in terms of the research method, based on decoupling analysis and the stochastic impacts by regression on population, affluence and technology (STIRPAT) model, this study explores the impact of a series of population development factors on carbon emissions, including population size, urban–rural structure, employment structure, age structure, population quality and population affluence, which is a supplement and extension of the existing research on the driving mechanism of carbon emissions. This will help decision makers formulate measures to improve population development and reduce emissions.

## 2. Literature Review

Researchers are interested in the study of carbon emission drivers in developing countries. Most of the current studies focus on carbon emission decomposition or decoupling from the perspective of energy consumption, economic development, technology progress and other factors [14,15,16,17]. To explore the interaction mechanism between these factors and carbon emissions, the Tapio decoupling model has proved to be an effective method and has been widely used. Shuai et al. investigated the global decoupling of economic growth and carbon emissions and concluded that high-incomes countries are more likely to have the expected decoupling relationship [18]. Zhang and Da analyzed the decoupling relationship between carbon emissions and economic growth in China, and the results indicated that economic growth is the primary driver of carbon emissions growth [19]. Song et al. utilized the decoupling model to evaluate the decoupling status and dynamic trends of carbon dioxide emissions at the provincial level in China [20]. The decoupling researches are also focused on the sector level, including construction, industry [21,22], product [23],transportation [24,25] and agriculture [26].

Economic output has become the main consideration in the study of carbon decoupling in China [27], and this consideration is also reflected in the research outside China [28]. However, the dependence of carbon emissions on other factors, such as population, has received little attention, and only a few studies have assessed the decoupling of carbon emissions from population-related factors. For example, Ma et al. explored the relationship between household carbon emissions and economic growth based on decoupling indicators, and concluded that household carbon dioxide emissions were in a weak decoupling state on the whole, and changes in CO_2_ emissions caused by population growth and economic growth were in a weak decoupling state and expansionary decoupling state, respectively [29]. The current research has also consistently found that population factors (i.e., size, growth and other parameters) are strongly correlated with carbon emissions [30,31,32,33]. Successful environmental social science research projects, such as the Infrastructure Project Assessment Tool (IPAT), place great emphasis on the relationship between population and the environment [34]. Therefore, in the transition period of population development, it is necessary to systematically understand the dependence of carbon emissions on population factors.

To clarify the interaction mechanism between population and carbon emissions, researchers examined the effect of various population factors on carbon emissions. Wang, et al. explored the impact of population size, per capita consumption, urbanization and an aging population on carbon emissions [11]. Zhu and Peng studied the impact of population change on China’s carbon emissions, and they revealed that consumption levels and population structure significantly affect carbon emissions [35]. Jorgenson and Clark argued that population size is positively correlated with carbon emissions [36]. More and more research argued that population size, population structure, quality and other indicators should be considered in the economy-environment model to fully reflect the impact of population factors on carbon emissions [37,38]. In some research, carbon emissions are related to population aging, and the working-age population is also considered an important indicator of future carbon emission mitigation [39,40]. Li et al. found that the relationship between the aging structure and carbon emissions in China can be described by an inverted U-shaped curve [41]. In some developing countries, population quality also has a significant impact on carbon emissions [42]. A cross-nation study adds to the discussion on the link between population size and other demographic factors and pollution, arguing that population increases are matched by proportional increases in emissions while a higher urbanization rate and lower average household size increase emissions [43].

In terms of population development characteristics, almost all of the important population factors, including population size, population structure, population quality and population distribution, are constantly changing, which have a complex and changeable impact on carbon emissions [35]. Generally, the impact of population on carbon emissions is uncertain due to the varied population features in different regions [44]. It is certain that if the population factors are measured by population scale, it cannot fully reflect the population impact on carbon emissions. However, this is what most studies have done when exploring carbon emissions drivers by multiple regressions in the economy-environment model. The assumption behind this treatment is that each individual in a population shares the same production and consumption behavior, but this assumption may be inaccurate and misleading [45]. Thus, an integral description that utilizes the multidimensional characteristics of population development is required, which will help to understand the effect of population on carbon emissions.

In summary, the impact of various population factors, including population size, population growth rate, age structure, urban–rural structure, employment structure, population quality, consumption structure and per capita GDP, on carbon emissions have been studied. Although some progress has been made, there are still some limitations, which highlight the following research gaps: (1) various population factors are simply used, and without an integral indicator to reflect multidimensional population development characteristics; (2) most studies on carbon decoupling have been typically conducted at a sectoral or country level and measured by economic outputs. This makes the relationship between population development and carbon emissions unclear.

To address the research gap, this study: (1) develops a population development index (PDI) to evaluate multidimensional population development; (2) establishes a decoupling model to investigate the decoupling between carbon emissions and the PDI in 30 Chinese provinces; (3) investigates the impact of various population factors on carbon emissions and explores policy suggestions to promote the decoupling of carbon emissions from PDI.

This paper is organized as follows. Section 3 describes the research methods. The study areas and the data sources are presented in Section 4, and Section 5 presents the results and discussion, which is followed by the final conclusions and policy implications.

## 3. Materials and Methods

### 3.1. PDI Construction

The PDI is developed through the following three steps: (1) select indicators that represent the level of population development; (2) determine the weight of each indicator; and (3) calculate the PDI.

#### 3.1.1. Indicator Selection

The evaluation of the population development level usually includes a series of complex index systems, including the total population index, the population structure index, population quality index, population economic activity index and these indexes objectively reflect the population development level [40]. Some important population studies provide reference for the establishment of a population development index [46,47,48,49,50]; population size, population growth, population quality, population living standard and population age structure are incorporated into the PDI in this study. The indicators that make up the PDI are shown in Table 1.

**Population size**: As a population factor that has a significant impact on carbon, it is often included in the IPAT/STIRPAT model to investigate the environmental pressure. In terms of population development, the more people there are, the more social wealth can be created. Population growth promotes the development of the service industry and industrial transformation [51], and also has a long-term positive impact on economic development [52]. In turn, economic growth promotes population development [53]. Under the current three-child policy in China, population size can be regarded as a positive indicator to promote population development. This study uses the total population and population growth rate to measure the population size.

**Population structure**: in this study, population structure is considered by age structure, urban–rural structure and employment structure.

In terms of age structure, it reflects the structural health of the population. The population aging trends will impose challenges for China’s sustainable development on the supply and demand sides in the long term [54]. Governments around the world are also urgently formulating policies to deal with the phenomenon of population aging [49,55]. Therefore, from the perspective of long-term population development, population aging is considered as a negative indicator in the PDI establishment.

Urban–rural structure reflects the distribution and migration of population in the process of urbanization. It is generally argued that the population distribution in urban areas is more concentrated, and the population density is higher than that in rural areas. The higher the urbanization level, the stronger the regional development. The level of population urbanization plays a very important role in promoting population progress and development [56], so the urban–rural structure is considered as a positive indicator of the PDI.

Employment structure: It is generally believed that the greater the number of people engaged in the secondary and tertiary industries, the higher the degree of development of industrial and social services. In terms of China’s current stage of development, the employment population in the secondary industry and tertiary industry will be considered as positive indicators of the PDI because both industry and service industry contribute significantly to economic and population development [57].

**Population quality**: population quality often represents the civilization construction level of a country or region, and is usually measured by the educational level of the population, which plays a very important role in promoting population development [58].

**Population wealth**: population wealth reflects the ability of the population to create wealth. The higher the per capita wealth is, the higher the people’s living standard and the higher the consumption level are. In the context of rapid economic development, the level of population wealth has further promoted the shift of population development to high quality [59].

#### 3.1.2. Weight Determination and PDI Calculation

All of the indicators are standardized to make different variables comparable by using the following formulas [60]:

(1) $$y_{+ij}=\left(x_{ij}-x_{ijmin}\right)/\left(x_{ijmax}-x_{ijmin}\right)$$

(2) $$y_{-ij}=\left(x_{ijmax}-x_{ij}\right)/\left(x_{ijmax}-x_{ijmin}\right)$$

where *y_+ij_* represents the positive indicator; *y_−ij_* represents the negative indicator; *x_ij_* represents the value of indicator *j* in province *i*; and *x_ijmax_* and *x_ijmin_* indicate the maximum and minimum value of the indicator *j*, respectively. Then, the entropy weight calculation is used to determine the importance of each indicator:

Firstly, to calculate the sample indicator weight:

(3) $$p_{ij}=x_{ij}/\sum_{i=1}^{n}x_{ij},$$

Secondly, to calculate the entropy of indicator j:

(4) $$e_{j}=-k\sum_{i=1}^{n}p_{ij}\times lnp_{ij},$$

(5) k=1/ln n

Thirdly, to calculate the utility value of each indicator:

(6) $$d_{j}=1-e_{j}$$

Finally, to calculate the indicator weight:

(7) $$w_{j}=d_{j}/\sum_{j=1}^{n}d_{j},$$

where *p_ij_* represents the share of province *i* on indicator *j*; *e_j_* is the entropy of indicator *j*; *n* is the number of the indicator; *d_j_* is the utility value of each indicator.

The linear weighted sum method is commonly used for evaluating the performance of a system which consists of multiple dimensions of indicators. By using this method, the performance value of the PDI in province *i*, can be calculated as follows:

(8) $$PDI_{i}=\sum_{j=1}^{n}w_{j}\times y_{ij},$$

where *w_j_* is the PDI weight of indicator *j*.

### 3.2. Carbon Emission Estimation

More than 95% of carbon emissions come from energy consumption [61], according to the IPCC National Greenhouse Gas Inventories and energy consumption [62]. The carbon emissions is calculated by following formula:

(9) $$C=\sum_{e=1}^{m}E_{e}f_{e}k_{e}\frac{44}{12},$$

where *C* represents the emissions; *m* is the number of the energy type; *E_e_* is the consumption of fossil fuel *e*; *f_e_* indicates the standard coal conversion factor of fossil fuel *e*; *k_e_* is the carbon emission factor for fossil fuel *e* [62,63]; and 44/12 is the molecular transition from carbon dioxide to carbon.

### 3.3. Decoupling Elasticity Model

The Tapio model proposed a theoretical framework of decoupling when studying the relationship between GDP, traffic volume and transport carbon emissions in the European Union, which has become a commonly used model to explore the correlation between social development and environmental impact [64]. The Tapio model described the GDP decoupling elastic of carbon emissions in transportation industry as:

(10) β=%ΔC/%ΔGDP

The decoupling model is improved in this study by using the PDI instead of a single index to reveal the decoupling features between population development and carbon emissions. The decoupling elasticity, *β*, can be represented as follows:

(11) $$\beta =E_{C}/E_{PDI}$$

(12) $$E_{C}=\Delta C/C_{b}=\left(C_{t}-C_{b}\right)/C_{b}$$

(13) $$E_{PDI}=\Delta PDI/PDI_{b}=\left(PDI_{t}-PDI_{b}\right)/PDI_{b}$$

where Δ*C* and Δ*PDI* represent the change of carbon emissions and the PDI from base year *b* to target year *t*, respectively; *C_t_* and *C_b_* denote carbon emissions in year *t* and year *b*, respectively; and *PDI_t_* and *PDI_b_* indicate the value of the PDI in year *t* and year *b*, respectively.

In order not to overinterpret slight changes as significant, a ±20% variation in the elasticity values around 1.0 is still regarded as coupling. Thus, coupling is defined as elasticity values of 0.8–1.2, and decoupling and negative decoupling is defined outside of this scope [64]. The decoupling can be divided into three degrees and eight states, as shown in Table 2.

### 3.4. The Extended STIRPAT Model

The STIRPAT model is introduced to evaluate the nonlinear impact of population, the economy and technological development on the environment [65,66]. It can be expressed as follows:

(14) $$I=aP^{b}A^{c}T^{d}e$$

(15) lnI=lna+blnP+clnA+dlnT+lne

where *I* represents the environmental impact, which is carbon emission in this study. *P* represents the PDI; *A* represents affluence per capita; *T* represents the technological level and is measured by technology market turnover and number of patents; *b*, *c* and *d* reflect the importance of *P*, *A* and *T* respectively; *a* and *e* are constants.

To identify the impact of population-related factors on carbon emissions, we disaggregated population factors into the following: population size (total and trend), population structure (age, urban–rural and employment structure), population quality (education level) and population wealth (economy and consumption). The extended STIRPAT model is expressed as follows:

(16) $$lnI=lna+b_{1}lnTP+b_{2}lnPG+b_{3}lnP_{0-14}+b_{4}lnP_{15-64}+b_{5}lnP_{65+}+b_{6}lnUR+b_{7}lnES+b_{8}lnET\;+b_{9}lnEP+b_{10}lnIR+b_{11}lnGP+b_{12}lnCE+dlnT+lne$$

where *A* is integrated into population wealth, other meanings are the same as the above.

In this study, time series data are used for the regression of 30 provinces to clarify the driving mechanism of carbon emissions in each province.

### 3.5. Ridge Regression

The standard form of multiple linear regression model is usually expressed as:

(17) Y=Xβ+ε,

where *Y* is a (*n* × 1) matrix of dependent variables, *X* is a (*n* × *p*) matrix (rank *p*) of independent variables, *β* is a (*p* × 1) vector of coefficients and *ε* denotes the normally distributed random errors. The unbiased estimate of *β* is normally given by:

(18) $$\hat{\beta }={\left(X^{T}X\right)}^{-1}X^{T}Y,$$

Due to the limitation of the social and economic variables, there are always correlations among the variables, that is, multicollinearity. When there is a multicollinearity between the independent variables, the $\;X^{T}X$ matrix is ill conditioned, i.e., the value of its determinant $\left|X^{T}X\right|$ ≈ 0. The calculation of the $X^{T}X$ matrix is sensitive to slight variations in the data. The addition or deletion of a variable or the slight change of a variable will have a great impact on the results, leading to the instability of the regression results. To control the general instability and inflation in estimating *β*, the ill-conditioned problem needs to be transformed into a conformity problem by adding a regularization term to the loss function, i.e., ridge regression [67]. The ridge regression model can obtain an acceptable biased estimate with small mean square error in independent variables through a bias variance tradeoff, which is one of the effective methods to deal with multicollinearity [68]. The general expression of the ridge regression model is as follows:

(19) $$\hat{\beta }={\left(X^{T}X+kE\right)}^{-1}X^{T}Y,$$

where *E* is unit matrix, *k* is the variable ridge regression coefficient in ridge traces and the value of *k* varies from 0 to 1. The ridge regression estimates are computed with various increasing values of *k*, starting from *k* = 0, until an optimum value of *k* is determined for where all the regression coefficients appear to have stabilized.

## 4. Study Areas and Data Sources

### 4.1. Study Areas

**Figure 1:**
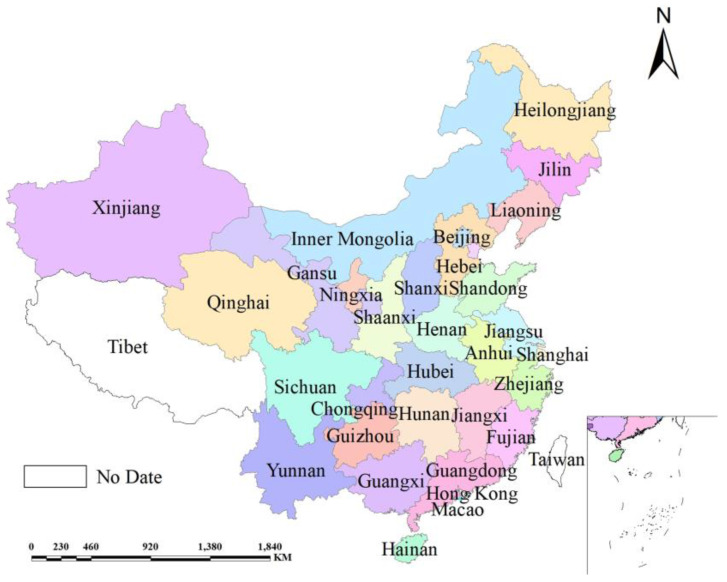
Study areas.

China’s 30 provinces (Figure 1) are investigated (excluding Tibet, Hong Kong, Taiwan and Macau where data are not available). These provinces have made great achievements driven by reform and an opening-up policy. For example, per capita GDP rose from less than CNY 10,000 in 2001 to more than CNY 60,000 in 2017, and the urbanization level has also improved significantly. However, there are significant differences between provinces. The economic strength and urbanization processes of the eastern provinces are higher than those central and western provinces, and the population distribution is also quite different.

The population density of the eastern provinces is much higher than that of the western provinces. The development status of different provinces and regions leads to differences in the spatial distribution of population development and carbon emissions. The decoupling state of carbon emissions and population development in different provinces may also vary greatly. In order to further clarify the decoupling state of each region and provide support for decision making, this study discusses the decoupling relationship between carbon emissions and the PDI at the national and provincial scales.

### 4.2. Data Sources

The PDI component data (2001–2017) are obtained from the China Statistical Yearbook and Population Census Bulletin. The energy consumption data (2001–2017) comes from the China Energy Statistics Yearbook. The data of Chinese administrative boundaries are obtained from the Resources and Environment Science and Data Center of the Chinese Academy of Sciences. These data sources are listed in Table 3.

## 5. Results and Discussion

### 5.1. PDI of the 30 Provinces in China

**Figure 2:**
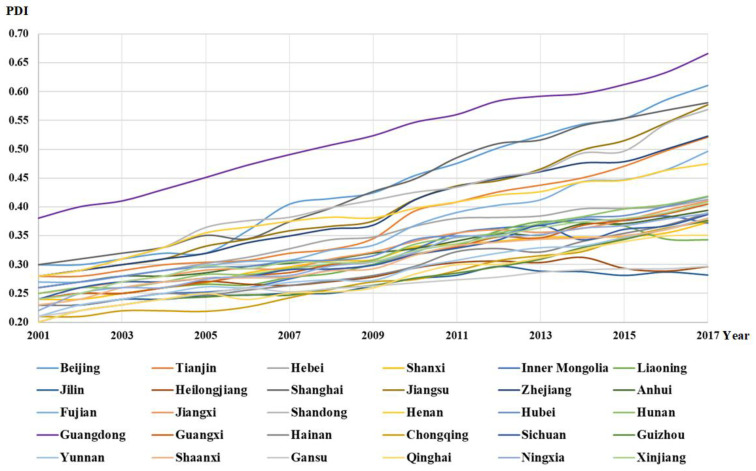
The PDI changes in 30 provinces from 2001 to 2017.

The PDI changes of 30 provinces from 2001 to 2017 are evaluated to reflect the characteristics of population development. Among the 30 provinces, Guangdong has the highest PDI score, as shown in Figure 2. As the province that contributes the most to China’s GDP, its per capita wealth is higher than that of the other 29 provinces, and the population age structure is getting younger. Beijing and Shanghai also have strong PDI competitiveness. Shandong, Jiangsu, Zhejiang, Tianjin, Henan and Fujian also saw a significant increase in the PDI between 2001 and 2017. These provinces have higher per capita wealth or population size, resulting in a higher PDI than other provinces.

Jilin, Heilongjiang and Gansu are at the bottom of the PDI list. Although continuous population urbanization is occurring in Jilin and Heilongjiang, the geographical location and climate problems of these two provinces have led to a large population outflow. In addition, the natural population growth rate is low, the population scale is on the decline and the elderly population continues to grow, which is not conducive to the long-term population and regional development. There is limited per capita wealth and an aging population, which are common characteristics of these three provinces. For these provinces, it is important to formulate relevant population policies to promote population inflow, reduce the proportion of the aging population and give full play to the dividend of population agglomeration so as to promote long-term population development.

The average PDI in the 30 provinces increases over time, while regional disparities are also reflected in the PDI, similar to how Table 4 shows, which contained the statistical information of the PDI. In 2001, Guangdong Province showed the optimal PDI of 0.38, while Guizhou and Qinghai had the lowest PDI, which is only 0.20, lower than the national average of 0.25. In 2017, the optimal PDI reached 0.67, while the lowest PDI was 0.28. The individual gap between the optimal PDI and the worst gradually widens each year in the sample period, indicating a huge development gap. Although the PDI in Hebei, Shanxi, Inner Mongolia, Anhui, Jiangxi, Hubei, Hunan, Guangxi, Hainan, Chongqing, Sichuan, Guizhou, Yunnan, Shaanxi, Gansu, Qinghai, Ningxia and Xinjiang showed a gradual upward trend, they still failed to reach the initial value of Guangdong, which indicates that there is great potential for further improvements in these provinces.

Reviewing the population development of all of the provinces, we find that Guangdong’s population wealth and population structure, regardless of age structure or employment structure, are in the best state in China. Therefore, for most provinces, improving the quality of population development should not only focus on accelerating the urbanization of population, but also pay attention to the improvement of population wealth and employment. Meanwhile, resources should be coordinated at the national level. Instead of widening the development gap gradually, provinces that develop first should lead those that develop later, and finally achieve common development.

### 5.2. Decoupling between Carbon Emissions and PDI

#### 5.2.1. Decoupling at the National Scale

The national decoupling between carbon emissions and the PDI during the study period can be divided into four states: expansive negative decoupling (EN), expansive coupling (EC), strong decoupling (SD) and weak decoupling (WD). Table 5 provides a complete decoupling relationship dynamic, showing that the interannual decoupling state gradually changes from EN to the decoupling state at the end of the study period. In the period from 2012 to 2015, each interannual decoupling both showed a SD state, while other periods, except from 2016 to 2017, showed a coupling state, including EN and EC. EN and EC reflects the close relationship between carbon emissions and the PDI, which indicates that both carbon emissions and the PDI increased, and carbon emissions changed more than the PDI in EN state. The decoupling state, including WD and SD, indicates that the dependence of carbon emissions on PDI decreases. In particular, in the SD state, the PDI continues to rise while carbon emissions are reducing, which is an ideal state.

In the early stage of the sample (2001–2007), economic globalization promoted China’s rapid development, and the economic growth rate reached over 9%, which also exacerbates the contradiction between social development driven by energy consumption and sustainable development. During this period, the carbon emissions growth rate is much higher than that of the PDI; EN is the primary decoupling state.

In 2007–2008 and 2008–2009, there was EC, the economic situation was not optimistic and the industrialization process slowed down due to the impact of the economic crisis. Industries with high energy consumption and high emissions, such as construction, had been largely shut down due to the decline in market purchasing power, which greatly reduced carbon emissions. Although the population unemployment rate increased and the growth rate of population wealth slowed down, from the perspective of multidimensional population development, the impact of the economic crisis on the population development is not obvious. As shown in Figure 2, the PDI of most provinces continued to grow during this period.

In each period between 2009 and 2012, the EN or EC showed that the link between carbon emissions and the PDI had strengthened after the economic crisis. In order to promote economic development and safeguard people’s well-being, China adopted a series of macro-economic regulation measures, including tax cuts and tax rebates, expanding domestic demand, etc., which led to the growth of carbon emissions. China also became the largest carbon emitter during this period [69]. In order to ease the pressure of carbon emissions, China paid more attention to the harmony with nature in the following years (2012–2017); SD was the main decoupling relationship during the period. However, it is important to note that EC also appeared during this period, which is a nondecoupling state. Our results show that the growth rate of the PDI is greater than zero in both decoupling years and nondecoupling years, but the change rate of carbon emissions in nondecoupling years is larger, while the change rate of carbon emissions in decoupling years is small or negative. In the long run, SD state may be difficult to maintain, which means that there is no real decoupling between carbon emissions and the PDI. The same decoupling trend is also reflected in the research of Shang and Luo [27]. Therefore, the key to decoupling is to effectively control the change rate of carbon emissions, while evaluating the effectiveness of the decoupling state requires examining the changes of carbon emissions and the PDI over a period, for example, taking 5 years as an evaluation cycle.

Table 5 also shows the long-term decoupling relationship. The results show that although the interannual decoupling relationship between 2011 and 2015 is dominated by SD, the tension between carbon emissions and population development is not actually alleviated from the five-year assessment period because EC is a long-term decoupling state. This means that the growth rate of carbon emissions is still higher than the growth rate of the PDI, and only shows a short and small decrease in some years, making the decoupling state vulnerable to variable changes in the short term. The change of the decoupling state undoubtedly shows that both active and passive emission reduction need a long-term process, and decoupling can only have a discernible effect on the climate if it is consistent over a number of years. The long-term change trend of the decoupling relationship from EN to EC and then to WD means that carbon emissions are gradually decoupling. However, WD also indicates that carbon emissions have not shown a downward trend, and there are still many efforts to be made at the national level, such as developing clean energy and improving population welfare, so as to achieve a stable strong decoupling state in some period in the future.

To further promote the decoupling of carbon emissions from the PDI, and prevent the transition from decoupling to coupling again, on the one hand, the national level should continue to adhere to the green and sustainable development, gradually eliminate the industries with high energy consumption and high emissions, and promote the transition from the consumption structure based on fossil energy to the utilization of renewable energy. On the other hand, the government should continue to create employment opportunities, improve the level of per capita education and the quality of the population, formulate population policies, improve the aging phenomenon and promote high-quality population development.

#### 5.2.2. Decoupling at the Provincial Scale

The interannual decoupling state of each province is determined by calculating the decoupling coefficients of each province from 2001 to 2017. The evolution trend of decoupling coefficient in most provinces is basically consistent with the national level. Meanwhile, the decoupling coefficient across most provinces also shows a similar trend without considering the decoupling state. However, the provincial interannual decoupling state includes not only the EN, EC, WD and SD state, but also the SN state. Ningxia, Qinghai and Gansu are significantly different. These provinces showed more SN state, indicating that the growth rate of carbon emissions is higher than that of the PDI, and there is unbalanced development between the two. The main reason is that these provinces, located in western China, are rich in fossil resources. In the context of the urgent need of local governments to improve their development level and the Great Western Development Strategy of China, the energy-driven development model will undoubtedly lead to a large amount of carbon emissions. Although the PDI has also improved, it also pays a high environmental cost (carbon emissions).

**Figure 3:**
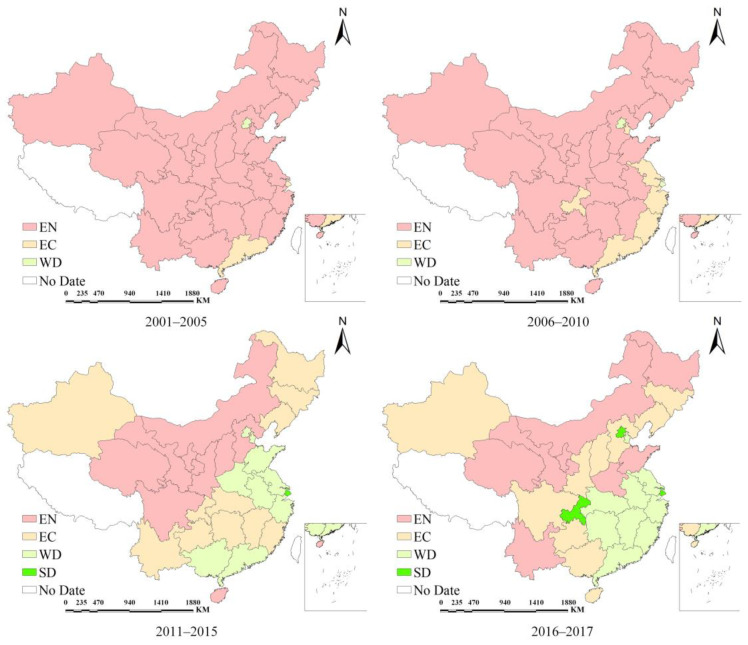
The decoupling evolution between carbon emissions and the PDI.

As mentioned above, a long-term state of decoupling might make more sense. The long-term evolution of decoupling is shown in Figure 3. The research period is divided into 2001–2005 (10th Five-Year Plan), 2006–2010 (11th Five-Year Plan), 2011–2015 (12th Five-Year Plan) and 2016–2017 (13th Five-Year Plan). It can be clearly seen that EN is the main decoupling state during 2001–2005 and 2006–2010, while EC state appeared in a few provinces and WD only appeared in Beijing and Shanghai. During the 12th Five-Year Plan period (2011–2015), more and more provinces began to show EC and WD states, and only Shanghai is in the SD state. The coexistence of EN, EC and WD is the main decoupling feature in this period. Most of the central and western provinces are in the EN state, indicating that carbon emissions and the PDI is still unbalanced, but the tension between carbon emissions and the PDI has eased compared with the previous two periods. Provinces in the EC state are mainly distributed in the central region, while most eastern coastal provinces are in the WD state.

In the 13th Five-Year Plan period (2016–2017), the number of provinces in the EN state has further decreased, and some provinces, including Beijing, Shanghai and Chongqing, appeared in the SD state, showing an ideal direction of decoupling evolution. However, there are also some provinces that showed the opposite direction of evolution. For example, in the 12th five-year period, Shandong and Henan are in the WD state, Yunnan is in the EC state and from 2016 to 2017, the three provinces are in the EN state again. This may be due to the lack of complete data for the 13th Five-Year Plan (2016–2020). As mentioned above, the short-term decoupling relationship is susceptible to the effects of variable tiny changes. Still, it is a reminder to managers that they need to continue to reduce carbon emissions as they develop to prevent carbon emissions from rising again.

From the perspective of the decoupling evolution of each province, we found some noteworthy phenomena. The decoupling status of some provinces, including Inner Mongolia, Gansu, Ningxia and Qinghai, did not changed during the four periods, which is more reflected in EN. In terms of geographical location, these provinces are all located in the central and western regions of China. Due to the population flow, especially some young labor force to the eastern provinces, there are obvious differences in population development between these provinces and eastern provinces of China. In addition to geographical conditions, economic policies and other reasons, although the PDI of each province is on the rise, the development gap between regions is gradually widening, which is consistent with the results of Section 5.1. Meanwhile, these provinces are key players in China’s power grid supply. Due to the large population and high energy demand of central and eastern provinces, coupled with the resource mismatch between provinces in China, the stable supply of electricity requires the export of resource-rich provinces, such as Inner Mongolia, Gansu, Qinghai and Ningxia [70].

The existing studies show that the power sector is one of the largest carbon emission sectors in China [71]. If China’s power sector was considered as a country, it would be the third largest carbon emitter in the world [72,73]. However, the embodied carbon emissions associated with power transfer are not considered in our study. Large-scale electricity production generates carbon emissions locally, so decoupling remains a challenge for these provinces. The decoupling changes of Henan and Shandong are also worth paying attention to because their decoupling states have undergone a transition from coupling to decoupling and then to coupling. To further promote the decoupling of carbon emissions, two major efforts may be possible: on the one hand, optimize the energy production structure and gradually replace the current coal-dominated secondary power generation structure; on the other hand, formulate policies to attract talent and improve the population welfare to promote the improvement of the PDI.

### 5.3. Analysis of Population Effect on Carbon Emissions

A ridge regression is used to eliminate the influence of multicollinearity among the variables on the regression results. Supported by time series data, the extended STIRPAT model results of 30 provinces are obtained, as shown in Table 6. For each of the 30 provinces, the regression equation is significant (*F* statistic sig < 0.05), and the fitting degree (*R^2^* ≥ 0.9) is good. However, some of the variables in some provinces do not pass the significance test of the ridge regression with 90 percentile confidence intervals, for example, *PG* in Tianjin, *P_65+_* in Hebei and other specific significance results are also presented in Table 6.

The STIRPAT model is utilized to explore the impact of different population factors on carbon emissions, and on this basis to explore policy recommendations to promote decoupling. From our empirical results, we identify several meaningful phenomena.

First, compared with other factors, population growth has no significant impact on carbon emissions in most provinces and the total population has an impact on carbon emissions in all of the provinces. As one of the main driving factors of carbon emissions, the total population promotes the growth of carbon emissions in most provinces, while the inhibiting effect is only in a few provinces (Liaoning, Jilin, Heilongjiang, Anhui, Hubei and Chongqing). For these provinces that have the effect of population inhibiting carbon emissions, attractive talent introduction policies can be formulated to promote population transfer, further play the emission reduction effect of population and promote decoupling between carbon emissions and the PDI. In addition, according to our results, controlling rapid population growth is obviously beneficial to carbon emission reduction in most provinces, but it should be noted that it may accelerate the emergence of other social problems, such as the phenomenon of population aging. The results show that the aging phenomenon in most provinces promoted the growth of carbon emissions, which means the carbon emissions are not mitigated and is not conducive to population development.

Second, the labor-oriented age structure contributes to the growth of carbon emissions, and the aging population is negatively correlated with carbon emissions in provinces with a higher PDI, while positively correlated with carbon emissions in provinces with a lower PDI. This is consistent with the results of Zhang and Tan [40]. Even after retirement, as the older individuals continue to look for other jobs, the swelling labor force led to the growth of carbon emissions. In addition, they are less willing to pay for environmental protection because the costs are immediate, but they will not benefit from a high-quality environment in the future. It may be helpful to promote carbon decoupling by build more green leisure places for the elderly to ease the labor glut.

Third, the obvious improvement of the urban–rural structure means that economic development is effective, and the population is richer and has a stronger purchasing power. On the one hand, the improvement of the living standard lead to more direct and indirect carbon emissions, including more direct energy demand and fuller range purchases of home appliances, as well as more entertainment and leisure spending.

On the other hand, a higher industry and technology level promotes the consumption of more commodities and stronger purchasing power, and demand further promotes the development of the industrial and technological level. Many are choosing to shift from agriculture to higher-paying secondary and tertiary industries. The changes in employment structure also have an impact on carbon emissions, and the employment in both secondary and tertiary industries has contributed to the growth of carbon emissions during the current development period in almost all of the provinces. This means that the development level of China’s tertiary industry still needs to be improved because, theoretically, the more people engaged in the tertiary industry, the more developed, cleaner and more efficient the tertiary industry will be, and the lower the carbon emissions will be.

Therefore, in order to promote the decoupling of carbon emissions from the PDI, it is necessary to establish low-carbon supporting industries based on the characteristics and needs of the provinces. For example, for those provinces that are in the SN decoupling state, most of which are in central and western China, they can make use of their location and resource advantages to vigorously develop wind power, natural gas, new energy and other industries to shift the way of people’s life towards reliance on clean energy. This is not only conducive to promoting the decoupling of carbon emissions but is also conducive to optimizing the employment structure. While promoting China to achieve the carbon peak and carbon neutral goals, it will also raise the level of population development.

At last, population quality contributes to carbon emissions, although it is not significant in some provinces. Studies have shown that education is positively correlated with carbon emissions [74,75]. In China, improving population quality can promote economic prosperity, which, in turn, contributes to more carbon emissions. High-quality people also tend to have the ability to do more consumption and other behaviors that contribute to carbon emissions [76,77,78]. Environmental protection, therefore, should be integrated into the existing teaching system as a classroom teaching content. The government should guide people to adopt a green and low-carbon consumption pattern, such as introducing free buses to replace private cars, so as to promote the change of population’s consumption concept and promote carbon decoupling. This is not only for the provinces in SN decoupling state, but also for other provinces in China.

## 6. Conclusions

As working towards sustainable population development is an important part of carbon mitigation efforts, this study conducts a decoupling relationship analysis between carbon emissions and the PDI and investigates the influential mechanism between them. The following objectives are achieved in this study: (1) an integral population-related indicator, the PDI, is constructed to reflect the population development features, including population size, age structure, urban–rural structure, employment structure, population quality and personal wealth; (2) the decoupling model is established to investigate the decoupling relationship between carbon emissions and the PDI; and (3) the impact of population factors on carbon emissions are investigated and some suggestions are put forward for promoting carbon decoupling. The main findings and policy implications are as follows:

There is a significant increase in the PDI in all of the provinces, however, the inter-provincial gap has widened in terms of population development. In order to narrow the gap, the local governments should pay attention to the multidimensional population development process, and the central government should the coordinate resources and talent to favor China’s western provinces.

The decoupling relationship between carbon emissions and the PDI at the national level has experienced a transition from EN to EC, and then to the decoupling state, showing an ideal evolution process. The decoupling degree at the provincial level has also strengthened from 2001 to 2017, but some provinces are still in the EN state. These provinces can promote the decoupling of carbon emission from the PDI by developing clean energy supporting industries and increasing subsidies for clean energy markets.

The influence of population factors on carbon emissions is different in different provinces, but the total population, population wealth, population urbanization, labor force population and elderly population in most provinces are almost always positively correlated with carbon emissions. To promote the decoupling of carbon emissions from the PDI, provinces should develop low-carbon-supporting industries according to their own characteristics.

Although our research is focused on China, given that it is the world’s largest carbon emitter and most populous country, this study may help to prompt managers to focus on sustainable population development, not just high-quality economic development, as China shifts to high-quality development. Meanwhile, these implications may also promote some studies on population decarbonization in other countries of the world, thus promoting sustainable human development at the international level. In addition, due to the complexity of the influencing factors of carbon emissions, decoupling research can be further extended to other factors in the future, so as to promote the development of overall decoupling.

Still, this study also has limitations: On the one hand, in the construction process of the PDI, this study only focused on several major aspects of the current population, which can represent the development degree of population to some extent, but it is not comprehensive from the perspective of all-round evaluation. On the other hand, the carbon emission accounting in this study is based on the end-energy consumption of each province. Given the large-scale electricity trade at the provincial level in China, this will lead to a large amount of embodied carbon transfer, which is not considered in our study. In future studies, we will explore more comprehensive indicators of population development and explore the possible impact of embodied carbon transfer on decoupling.

## Figures and Tables

**Table 1:** PDI indicator.

Index	Sub-Indicator	Data Interpretation	Symbol
Size	Total regional population	Total population	TP
	Demographic trends	Natural population growth rate	PG
Structure	Age structure	Population aged 0–14	P_0–14_
		Population aged 15–64	P_15–64_
		Population over 65 years old	P_65+_
	Urban–rural structure	Population urbanization rate	UR
	Employment structure	Employed population in the secondary industry	ES
		Employed population in the tertiary industry	ET
Quality	Education level	Higher education proportion	EP
		Adult illiteracy rate	IR
Wealth	National economic output	GDP per capita	GP
	Consumption ability	Per capita consumption expenditure	CE

**Table 2:** Decoupling degrees.

Degree	State	Symbol	ΔC	ΔP	β
Decoupling	Strong decoupling	SD	<0	>0	<0
	Weak decoupling	WD	>0	>0	0 < t < 0.8
	Recessive decoupling	RD	<0	<0	>1.2
Coupling	Recessive coupling	RC	<0	<0	0.8 < t < 1.2
	Expansive coupling	EC	>0	>0	0.8 < t < 1.2
Negative decoupling	Strong negative decoupling	SN	>0	<0	<0
	Weak negative decoupling	WN	<0	<0	0 < t < 0.8
	Expansive negative decoupling	EN	>0	>0	>1.2

**Table 3:** Data sources.

Data	Data Description	Year	Source
Population size	Total population and growth trend	2001–2017	China Statistical Yearbook
Population structure	Urban–rural structure, age, labor force distribution	2001–2017	China Statistical Yearbook; The data for 2010 are from the sixth National Population Census Bulletin
Population quality	Education level	2001–2017	China Statistical Yearbook
Personal wealth	Affluence degree	2001–2017	China Statistical Yearbook
Energy consumption	Fossil energy consumption	2001–2017	China Energy Statistical Yearbook
Boundaries	Chinese provincial administrative boundary	2015	Resources and Environment Science and Data Center, Chinese Academy of Sciences

**Table 4:** The PDI Statistical information.

Year	Maximum	Minimum	Max-Min	Average
2001	0.38	0.20	0.18	0.25
2002	0.40	0.21	0.19	0.25
2003	0.42	0.21	0.21	0.26
2004	0.43	0.21	0.22	0.27
2005	0.45	0.22	0.23	0.29
2006	0.47	0.23	0.25	0.30
2007	0.49	0.24	0.25	0.31
2008	0.51	0.25	0.26	0.32
2009	0.52	0.26	0.26	0.32
2010	0.56	0.27	0.29	0.35
2011	0.57	0.27	0.29	0.36
2012	0.58	0.28	0.31	0.37
2013	0.59	0.29	0.31	0.38
2014	0.59	0.29	0.30	0.39
2015	0.61	0.28	0.33	0.40
2016	0.63	0.29	0.35	0.41
2017	0.67	0.28	0.38	0.43

**Table 5:** Decoupling trends at the national scale from 2001 to 2017.

Period	ΔC	ΔP	Decoupling Elasticity	Decoupling Degrees
2001–2002	>0	>0	4.21	EN
2002–2003	>0	>0	3.72	EN
2003–2004	>0	>0	2.96	EN
2004–2005	>0	>0	2.60	EN
2005–2006	>0	>0	2.27	EN
2006–2007	>0	>0	1.53	EN
2007–2008	>0	>0	1.02	EC
2008–2009	>0	>0	0.99	EC
2009–2010	>0	>0	2.07	EN
2010–2011	>0	>0	1.38	EN
2011–2012	>0	>0	1.13	EC
2012–2013	<0	>0	−0.15	SD
2013–2014	<0	>0	−0.22	SD
2014–2015	<0	>0	−0.08	SD
2015–2016	>0	>0	1.14	EC
2016–2017	>0	>0	0.77	WD
2001–2005	>0	>0	2.94	EN
2006–2010	>0	>0	1.99	EN
2011–2015	>0	>0	1.19	EC
2016–2017	>0	>0	0.77	WD

**Table 6:** Extended STIRPAT model results for 30 Chinese provinces.

Province	lnTP	lnPG	$\ln P_{0-14}$	$\ln P_{15-64}$	$\ln P_{65+}$	lnUR	lnES	lnET	lnEP	lnIR	lnGP	lnCE	lnT	*Cons*	*R^2^*
Beijing	0.589 **	0.004 **	−0.233 ***	0.682 **	−0.575 **	0.62 ***	0.527 ***	0.419 **	0.069 *	0.001	−0.156 ***	1.548 **	−0.934 **	1.415 ***	0.95
Tianjin	1.033 **	0.002	−0.13 *	0.837 *	−0.254 *	1.458 **	0.779 ***	−0.35 *	0.106 **	0.003 *	0.215 **	0.746 ***	−0.378 **	2.566 ***	0.93
Hebei	0.891 **	0.006 *	−0.018 **	0.445 *	−0.046 *	0.734 ***	0.433 **	0.217 ***	0.405 **	0.005 *	0.768 **	0.371 ***	−0.199 ***	−1.054 ***	0.94
Shanxi	0.465 ***	−0.003 **	−0.007 *	0.624 **	−0.087 ***	0.695 ***	0.352 ***	0.423 ***	0.618 **	0.011	0.836 ***	0.262 **	−0.107 **	3.45 ***	0.97
Inner Mongolia	0.562 ***	0.016	0.187 *	1.396 ***	0.024 **	0.339 ***	−0.195 ***	0.28 **	0.525 *	0.021	0.787 **	−0.543 ***	0.152 ***	−2.645 ***	0.96
Liaoning	−0.526 **	−0.158 **	−0.122 *	−0.263 **	0.394 ***	0.637 **	−0.119 ***	0.135 ***	0.176 *	0.032 *	0.387 ***	0.214 *	0.103 *	3.699 ***	0.97
Jilin	−0.874 ***	−0.079 *	−0.138 **	−0.462 *	0.191 **	0.89 ***	−0.273 ***	0.105 *	0.254 **	0.177	0.344 ***	−0.176 ***	0.462 ***	2.67***	0.92
Heilongjiang	−0.479 **	−0.165 *	−0.241	−0.015 ***	0.452 **	0.746 ***	−0.172 ***	0.078	0.125 **	0.084 *	0.92 ***	0.256 ***	0.201 **	0.983 ***	0.94
Shanghai	0.342 *	0.31 **	0.385 ***	0.874 ***	−0.653 *	0.839 **	0.548 ***	−0.461 **	0.11 **	0.021	0.104 ***	0.356 ***	−1.529 ***	−3.607 ***	0.91
Jiangsu	0.835 **	0.024 *	−0.426 **	0.262 ***	−0.115 ***	0.56 ***	0.137 ***	0.268 ***	−0.417	0.043 *	−0.181 ***	−0.134 **	−0.296 ***	−0.425 **	0.96
Zhejiang	0.943 **	0.16 *	−0.227 **	0.396 **	−0.241 ***	0.567 ***	0.253 ***	−0.275 ***	0.272 **	0.05	0.174 ***	0.233 ***	−0.652 **	−0.782 ***	0.98
Anhui	−0.684	0.037 ***	−0.196 *	−0.125 **	0.087 *	0.239 ***	0.192 ***	−0.247 *	0.015 *	0.027 *	0.865 **	0.223 **	0.314 ***	−1.654 ***	0.98
Fujian	0.572 ***	0.186 *	0.359	0.06 ***	−0.254 **	0.304 **	0.131 ***	0.107 ***	0.225 ***	0.011	0.148 ***	0.435 ***	−0.552 ***	−2.645 ***	0.96
Jiangxi	0.631 **	0.104 *	−0.165 **	0.047 **	−0.129 **	0.402 ***	0.113 ***	0.147 ***	0.093 **	0.032 *	0.106 ***	0.264 *	−0.118 *	−3.135 ***	0.95
Shandong	1.014***	0.037 **	0.562 **	0.106 **	0.191 ***	−0.148 ***	0.105 **	0.174 ***	0.229 *	−0.057 *	0.133 ***	−0.088 ***	−0.325 ***	−0.647 ***	0.99
Henan	0.108 **	−0.042	−0.117 ***	0.625 ***	−0.169 *	0.43 ***	0.241 ***	0.139 **	0.238 ***	0.074 *	0.201 **	0.196 **	−0.435 **	−0.342 ***	0.98
Hubei	−0.126 **	0.103 **	−0.182 *	−0.195 **	−0.084 *	0.763 ***	0.157 **	0.099 ***	0.335 **	0.025 **	−0.142 ***	0.254 ***	−0.373 ***	−2.051 **	0.96
Hunan	0.675 *	0.297	0.255	−0.356 **	0.217 **	0.193 ***	0.262 ***	0.108 ***	0.412 *	0.164 **	−0.057 **	0.106 *	−0.14 *	−1.956 ***	0.93
Guangdong	0.264 *	0.058	−0.173 **	0.426 ***	−0.113 ***	0.653 **	0.187 ***	−0.08 **	0.044 **	0.062 *	−0.158 ***	−0.043 ***	−0.384 ***	0.25 ***	0.94
Guangxi	0.392 **	0.006 *	0.075	0.101 ***	0.024 *	0.586 ***	0.195 ***	0.152 ***	0.325 *	−0.064 *	0.156 *	0.266	0.152 *	−3.615 ***	0.91
Hainan	0.345 **	−0.197	−0.161 *	0.874 **	−0.105 ***	0.406 ***	0.053 ***	0.076 ***	0.264 ***	−0.011	0.087 ***	0.092 ***	−0.093 ***	0.413 ***	0.95
Chongqing	−0.27 **	0.155	−0.126 **	0.154 ***	0.219 ***	0.513 *	0.268 ***	0.027 **	0.112 **	0.174	−0.215 *	−0.139 ***	−0.248 **	1.158 ***	0.95
Sichuan	0.265 ***	−0.046 *	−0.142 *	0.349 ***	0.134 **	0.409 ***	0.115 *	0.047 ***	0.025 ***	0.031	0.157 ***	0.136 ***	−0.154 ***	−0.974 ***	0.96
Guizhou	0.437 **	−0.169 ***	−0.159 *	0.264 **	0.101 ***	0.223 ***	0.295 ***	0.154 **	0.132**	0.227**	0.076 ***	0.084 ***	−0.106 ***	−1.482 ***	0.98
Yunnan	0.168 *	−0.049	−0.127	0.351 *	0.145 *	0.413 ***	0.126 **	0.173 ***	−0.009 ***	0.242 *	0.175 ***	0.153 ***	−0.215 ***	−0.186 ***	0.93
Shaanxi	0.582 ***	0.036 **	−0.103 **	0.517 **	0.089 **	0.494 **	0.164 ***	0.055	0.081***	0.107	0.187 **	0.243 ***	−0.254 *	−2.413 **	0.98
Gansu	0.411 **	−0.15 *	−0.187 ***	0.234 **	0.073 *	0.513 ***	0.195***	0.154 *	0.102 **	0.076 *	0.218 ***	0.106 *	−0.182 ***	−1.181 ***	0.94
Qinghai	0.365	0.064 **	−0.077 *	0.316 ***	0.122 **	0.435 ***	0.207**	0.085 ***	0.039 *	0.106	0.159 **	0.193 **	−0.25 **	−1.647 ***	0.91
Ningxia	0.284 **	−0.091 *	−0.111 ***	0.223 **	0.067 **	0.369 **	0.098**	0.027 ***	0.115 ***	0.17 **	0.151 ***	0.185 ***	0.086***	−3.812 **	0.95
Xinjiang	0.162 **	0.054 *	0.138 **	−0.435 ***	0.1	0.62 ***	0.195***	0.07 *	0.184 **	0.139 **	0.159 ***	0.187 *	−0.164 **	1.643 ***	0.97

Note: ***, ** and * represents significant at 1%, 5% and 10% levels, respectively.

## Data Availability

The data used in this study can be obtained from the China Statistical Yearbook, the China Energy Statistical Yearbook and the provincial statistical yearbook.
